# Inequality and support for government responses to COVID-19

**DOI:** 10.1371/journal.pone.0272972

**Published:** 2022-09-21

**Authors:** Hai-Anh H. Dang, Edmund Malesky, Cuong Viet Nguyen

**Affiliations:** 1 Data Production and Methods Unit, Development Data Group, World Bank, Washington, DC, United States of America; 2 International School, Vietnam National University, Hanoi, Vietnam; 3 Indiana University, Bloomington, IN, United States of America; 4 IZA, Bonn, Germany; 5 Duke University, Durham, NC, United States of America; 6 Mekong Development Research Institute, Hanoi, Vietnam; Sam Houston State University, UNITED STATES

## Abstract

Despite a deep literature studying the impact of inequality on policy outcomes, there has been limited effort to bring these insights into the debates about comparative support for government responses to the COVID-19 pandemic. We fill this gap by analyzing rich survey data at the beginning of the pandemic in April 2020 from six countries spanning different income levels and geographical locations—China, Italy, Japan, South Korea, the United Kingdom, and the United States. We find that poorer individuals are less supportive of government responses. Furthermore, poorer individuals residing in more economically unequal countries offer even less government support. We also find that both economic and non-economic factors could affect the poor’s decisions to support stringent government policies. These findings suggest that greater transfers to the poor may offer an option to help increase support for strict policies and may reduce the potential deepening of social inequalities caused by the pandemic.

## Introduction

A recent *Lancet* article suggested that fatalities are lower and economic recovery faster in countries that chose more stringent policies to combat COVID-19 [1]. However, the analysis does not address why these countries were able to implement stricter economic and social lockdowns on their citizens, while other countries either chose to avoid such closures, or were unable to effectively implement and enforce restrictive measures. While regarded as essential in the fight against the pandemic, government responses have varied widely across countries, at least during the initial reactions [2, 3], and these differences appear to be associated with success in limiting mobility, enforcing social distancing, and ultimately infection and fatality rates [4, 5].

In this paper, we suggest that during a time of crisis, policy responses to the pandemic—including voluntary measures such as self-quarantines, wearing a mask, and vaccination—require strong support from all population groups to be effective. Nevertheless, few studies exist that test whether the richer and the poorer share similar levels of support for such government responses. We fill this gap by analyzing a six-country survey covering different income levels and geographical locations—China, Italy, Japan, South Korea, the United Kingdom, and the United States—in April 2020, when most countries were facing their first surge in infections and designing their response efforts. In particular, we argue that underlying levels of inequality were critical in constraining officials’ choices when rapid responses could potentially thwart disaster.

### Inequality and pandemic response

There is a deep literature in political economy studying the endogenous relationship between economic inequality and policy making. Clearly, choices that governments make regarding taxes, social welfare, immigration, and economic integration have differential effects on citizens and can lead to divergence in economic fortunes [6, 7]. On the other hand, inequality limits the space for policy options and severely hampers policy implementation [8]. Some citizens, due to their position in society and level of resources, may be either unwilling or unable to agree to government directives or abide by government regulations. Empirically disentangling this relationship is extremely difficult, because of the interplay between inequality and policy developments over time. Original institutions and policy choices shape the distribution of resources in society, allocating political power to some actors, who then use it to influence future institutional and policy choices [9, 10].

Thus far, the balance of the literature has been on demonstrating how institutions affect inequality, but there is less evidence on how deep-seated structural inequality influences policy choices. Scholars have shown, however, that inequality is associated with lower levels of public good provision [11, 12], including in healthcare [13]. At the individual level, research demonstrates that high levels of inequality reduce trust in government institutions, undermining policy compromises and implementation [14]. Citizens may be unwilling to make sacrifices if they are not convinced that authorities will compensate them for their efforts [15, 16]. Lower levels of education among poorer groups may exacerbate distrust, when the relationship between their sacrifice and country-level policy goals are not sufficiently clear [17]. Related work on marginalized groups, which is associated with inequality, has shown that marginalized individuals are less trusting of government and less likely to comply with public health advisories [18–20].

The impact of inequality on policy choices is most critical in *hard times*, such as war, financial crises, or pandemics, when government leaders must quickly respond to the threat, but some individuals are either unwilling or unable to abide by government strictures [21, 22]. They may see the policies as placing an unfair burden on them relative to richer individuals, lack trust in the policy motivations, or the necessary measures may simply not be affordable due to low levels of wealth and savings, making it difficult to cushion the blow with consumption smoothing [23–25]. Inequality also undermines societies’ social fabric during such periods, creating group conflict and complicating coordinated efforts to combat health crises [26, 27].

It is now quite clear that the severity of the COVID-19 pandemic differed dramatically across individual countries; however, debate remains about what factors are most responsible for the variance [28]. Disagreement is particularly contentious regarding the effectiveness of policy responses. While some countries were able to impose restrictive economic lockdowns on their populations and reduce infection and ultimately fatality rates, other countries either chose to avoid such closures, or were unable to effectively implement and enforce restrictive measures [3–5].

Inequality has been an important part of policy debates over COVID-19 responses measures. Poorer citizens were more likely to work in service sector jobs, such as restaurants and retail, which were the immediate victims of economic lockdowns. They had less accumulated savings and therefore were less likely to afford extended time away from work. They were also less able to transition to virtual work, either because of the nature of their occupation or because they lacked sufficient space and internet access [29]. Classification of many poorer workers as “essential employees” in super markets and delivery services meant that they were more likely to be exposed to the disease [30]. Shutdowns of public services had a more severe impact on poorer communities, who were more likely to rely on public transportation and less likely to have private child care options. Among the very poorest, school closures not only endangered education prospects but also deprived children of free breakfast and lunches. In the United States, fine-grained data have been used to demonstrate a K-shaped effect of COVID-19, where richer populations reduced expenditures and increased their income, while poorer citizens actually increased their expenditures as their income declined [31].

We thus suggest the following hypotheses.

*H1*: *Citizens in lower income strata were more likely to resist strict policies under government orders than those in higher income strata*.
*H2*: *The discontent among poorer income strata is likely more severe in countries with higher levels of inequality*.

Once we can establish the validity of these two hypotheses, we go one step further and examine the underlying motivations for the relationship between income levels and support for government policies. Given our earlier review of the literature, we posit the next hypothesis.

*H3: Poorer citizens were particularly afraid of the economic burden they would face and, to a lesser extent, non-economic factors (such as limits to leisure and family time)*.

### Some illustrative macro-evidence on inequality and government policy stringency

**Fig 1:**
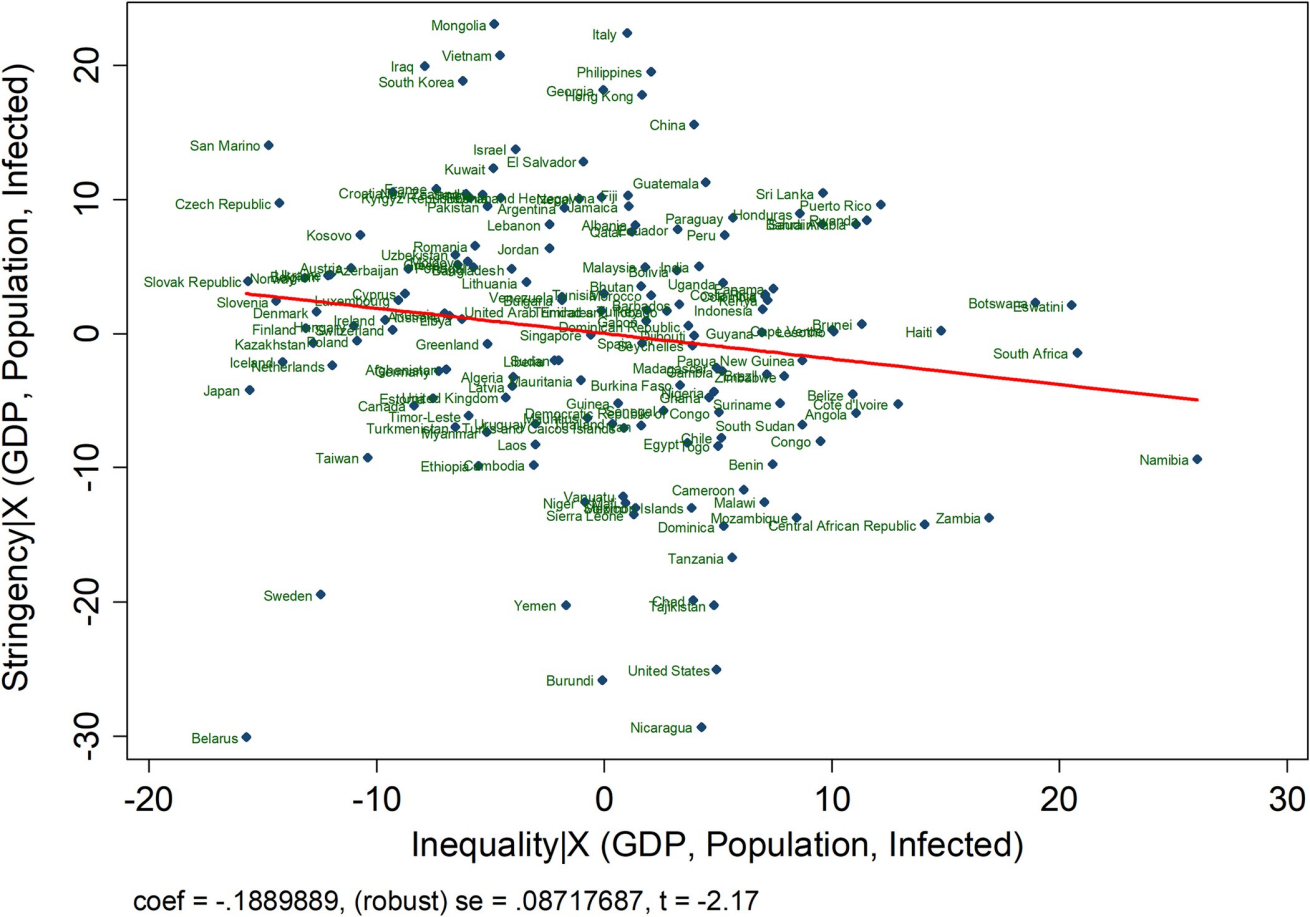
Adjusted relationship between inequality and policy stringency from February 1, 2020 to April 14, 2020. This figure plots the relationship between stringency of country-level COVID-19 policies and inequality, controlling for countries’ population, GDP per capita, and exposure to the disease. Stringency is measured by the Oxford Covid Government Response tracker program, ranking countries’ daily policy on a standardized scale ranging between 0 (not stringent at all) to 100 (highly stringent). We limit the range to the period between February 1st, 2020 after the disease had already been discovered outside of China, but before April 14th, 2020, the day before our survey data was initiated. The index is comprised of seven indicators of lockdown, such as school closures and restrictions in movement, which are consistent with our survey questions below. We measure inequality using the SWIID database (Solt, 2019).

To illustrate the potential for these relationships, Fig 1 uses a partial regression plot to study the relationship between stringency of country-level COVID-19 policies and inequality, controlling for countries’ population, GDP per capita, and exposure to the disease. Stringency is measured by the Oxford Covid Government Response tracker program, ranking countries’ daily policy on a standardized scale ranging between 0 (not stringent at all) to 100 (highly stringent). We limit the range to the period between February 1^st^, 2020 after the disease had already been discovered outside of China, but before April 14^th^, 2020, the day before our survey data was initiated, and many countries were in the midst of their first surge and still designing their policy responses. The index is comprised of seven indicators of lockdown, such as school closures and restrictions in movement, which are consistent with our survey questions below. We measure inequality using the SWIID database [32], which is considered to be the most consistent measure of cross-national inequality. The figure provides tentative evidence that countries with higher inequality chose less restrictive policies in their initial response to the crisis.

While the correlation demonstrated in Fig 1 is illustrative and can help motivate further research, there is a clear ecological inference problem. We cannot tell from whether it is the poor that resisted stringent policy at the outset of the crisis and thereby drove the relationship or whether richer groups in society were pushing back against strict lockdowns, knowing their resources allowed them to self-protect. To further test our theory, we move toward more fine-grained survey on individual-level reactions to lockdown policies in six countries.

## Methods and data

We analyze individual data from a six-country survey to examine the extent to which citizens support their government’s responses to the COVID-19 pandemic. According to [33], data were collected by market research companies, including Lucid for Italy, UK and US, and dataSpring for China, Japan and Korea. The respondents were randomly selected from sampling frames that were available to these two companies. This survey was implemented between April 15 and April 23, 2020 by [33], covering 6,089 respondents from all geographical regions of China (22 regions), South Korea (16 regions), Japan (8 regions), Italy (20 regions), the United Kingdom (12 regions), and the four largest states in the United States (California, Florida, New York, and Texas). The survey was conducted online and the median response time for the survey was about 14 minutes.

The data for each country are nationally representative across age groups and gender [33]. The sample size hovers around 1,000 observations for each country, ranging from 963 for South Korea to 1,055 for the U.S. The survey contains information on basic demographic variables of respondents, their income (measured in quintiles), their self-reported assessments on the economic and non-economic consequences as well as their support of the government’s policy responses to the COVID-19 pandemic.

We compare the distributions of respondents by gender and age groups in the survey and the distributions of these variables derived from the official numbers in order to assess the survey’s representativeness at the national level (Table A.1 in the Appendix A of S1 File, which is similar to the results in [34]). The proportion of responders in each age group for Japan and the UK varies slightly. Yet, the differences do not appear to be substantial.

We further examine the distributions of respondents by income quintiles in Table A.2 in Appendix A of S1 File. The survey did not collect data on respondents’ precise incomes, but collected data on which of the five pre-COVID-19 income brackets (quintiles) they belong to. These income brackets are obtained by calculating quintiles of the gross household income distribution from the last available wave of nationally representative household surveys or census data, which capture the income distributions before the COVID-19 pandemic [33]. If the COVID-19 survey samples are representative of these income quintiles, the share of respondents in each quintile should equal 20%. Table A.2 in S1 File shows that the shares of respondents in each income quintile in the six countries are roughly close to 20%. However, these shares for the poorest two quintiles are statistically less than 20% for four countries, Italy, South Korea, the U.K., and the U.S., suggesting that the data may not allow us to clearly delineate differential impacts between these two quintiles.

An overall assessment of the government response to the pandemic is assessed with this question “Do you agree with the current approach taken by your government in response to the pandemic?” The respondents could select one of the five options “1 = Strongly disagree”, “2 = Somewhat disagree”, “3 = Neither agree nor disagree”, “4 = Somewhat agree”, and “5 = Strongly agree”.

Afterwards, survey respondents were asked to assess the effectiveness of the seven particular government measures, which correspond closely to the stringency index above.

- Shutting down schools
- Shutting down public transport
- Shutting down non-essential businesses
- Limiting mobility outside home
- Forbidding mass gatherings
- Introducing fines for citizens that don’t respect public safety measures
- Requiring masks to be worn outside by everyone

The specific survey question is “How effective do you believe each of these measures is in reducing the spread of the epidemic?” The respondents were asked to select one of the following five answer options “1 = Not effective at all”, “2 = Slightly effective”, “3 = Moderately effective”, “4 = Very effective”, and “5 = Extremely effective.” Table A.3 in Appendix A of S1 File presents the distribution of respondents’ answers to the government’s policy responses.

To enrich analysis, we collect our own data on COVID-19 infection rates at the region level (82 regions) for the six countries. The COVID-19 infection rate is measured as the number of cumulative COVID-19 cases over 1000 people in each region by April 14, 2020 (just before [33]’s survey started). The average COVID-19 infection rate is 1.04 per thousand, and ranges from 0.003 per thousand in Qinghai, China to 23.4 per thousand in New York, the U.S. For data on inequality, we primarily use data from SWIID database [32], but we also use data from the World Bank Development Indicators [35] for robustness checks. Table A.4 in Appendix A of S1 File reports the means of these variables for each country.

To examine the association between income inequality and support of the government’s responses to the COVID-19 pandemic, we estimate the following linear regression model with country fixed effects

$$Y_{ij}=\alpha +{Income\_Quintile}_{ij}\beta +X_{ij}\gamma +{Country}_{j}\delta +u_{ij}$$ (1)

where *Y*_*ij*_ is a dependent variable indicating support of the government responses of individual *i* in country *j*. The control variables, *X*, include individuals’ age and gender, urban residence, and the COVID-19 infection rate of regions. It should be noted that there is no information on ethnicity as well as education in the data set. There is some information on race (such as white, black, and other races), but for the U.S. sample only.

*β* represents the (vector of) coefficients of interest. Because of potential selection bias caused by omitted variables, we are unable to estimate the causals effect of the income quintiles. The estimates of *β* in Eq (1) thus reflect the association between the different income quintiles and individual assessment of government responses. We focus on the difference in assessment of the government responses between the poorer and the richer quintiles.

Further interacting the income quintile variables with a country’s inequality level (instead of using the country fixed effects dummy variable) allows us to probe into the hypothesis that the discontent among poorer income strata can be more severe in countries with higher levels of inequality. The coefficient of interest is *θ*_*L*_ in the following equation.


$$Y_{ij}=\alpha_{L}+{Income\_Quintile}_{ij}*{Country\_Inequality}_{j}\theta_{L}+{Income\_Quintile}_{ij}\beta_{L}+X_{ij}\gamma_{L}+\omega_{ij}$$ (2)

To shed more light on the mechanisms of impact, we test whether economic and non-economic factors mediate the effects of income on support for government responses using the standard mediation approach [36]. We first regress individuals’ self-reported assessment of several economic and non-economic consequences (mediator variables) on the income quintiles.

$$M_{ij}=\alpha_{M}+{Income\_Quintile}_{ij}\beta_{M}+X_{ij}\gamma_{M}+{Country}_{j}\delta_{M}+\varepsilon_{ij}$$ (3)

where *M*_*ij*_ is a mediator variable of interest. The lower subscript *M* indicates the parameters in the mediator regression.

We subsequently regress individuals’ overall assessment of the government response on the income quintiles and mediator variables.


$$Y_{ij}=\alpha_{Y}+{Income\_Quintile}_{ij}\beta_{Y}+M_{ij}\theta_{Y}+X_{ij}\gamma_{Y}+{Country}_{j}\delta_{Y}+v_{ij}$$ (4)

The lower subscript *Y* indicates the parameters in the outcome regression, which controls for the mediator. The indirect effect is estimated by the product of *β*_*M*_ and *θ*_*Y*_ (from Eqs (3) and (4) above). We can compute the indirect effect as a percentage of the total effect, that is, (*β*_M_*θ_*Y*_)*100%/*β* for each income quintile (*β* is the vector of coefficients on *Income*_*Quintile* in Eq (1)). We focus on estimating the indirect effect of the poorest income quintile on the overall assessment of government responses through several economic and non-economic outcomes that are caused by the pandemic. Put differently, the indirect effects with these outcomes can help better explain the channels through which being in the poorest quintile leads to less support for government responses. These results can help shed light on the third hypothesis discussed earlier.

### Poorer quintiles are less supportive of government responses

**Fig 2:**
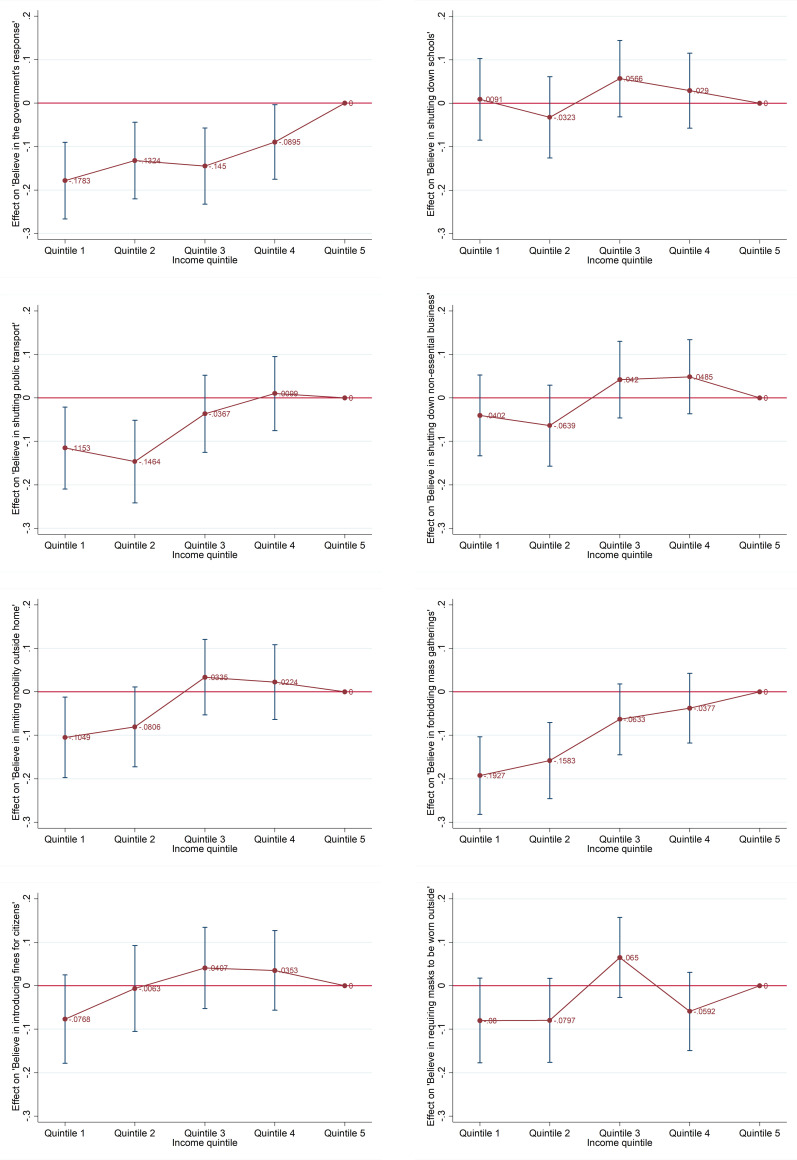
Estimated effects of income inequality on agreement with government’s responses to the COVID-19 pandemic (by policy). This figure reports the coefficients of the income quintile of belief that their government’s responses to the COVID-19 pandemic was effective. Quintile 5 (richest income quintile) is used as the reference category. Control variables include age groups, gender, urban dummy, country dummies, and COVID-19 infection rates. The full regression results are presented in Table A.1 in Appendix A of S1 File. Panel A. Believe government response to the pandemic is effective. Panel B. Believe shutting down schools is effective. Panel C. Believe shutting down public transport is effective. Panel D. Believe shutting down non-essential businesses is effective. Panel E. Believe limiting mobility outside home is effective. Panel F. Believe forbidding mass gatherings is effective. Panel G. ‘Believe in introducing fines for citizens that don’t respect public safety measures.’ Panel H. ‘Believe in requiring masks to be worn outside by everyone.’

We analyze individual data from a six-country survey to examine the extent to which citizens support their government’s responses to the COVID-19 pandemic (see Methods section for discussion on the data and estimation method). Fig 2 presents our estimation results of citizens’ assessments of eight government responses to the pandemic (see Appendix A, Table A.5 in S1 File for full regression results). Overall, individuals in the four lower income quintiles are less supportive of strict government responses (Fig 2, panel A) than those in the richest (income) quintile (the reference category). The differences are statistically significantly different at the five percent level or lower. Furthermore, the poorer quintiles tend to be less supportive than the richer quintiles. The poorest quintile is 0.18 points (or 4.8%) less supportive than the richest quintile (the largest magnitude on the 1 to 5-point scale), followed by the second poorest quintile and middle quintile (0.13 and 0.15 less supportive respectively), and the second richest quintile (0.09 points less supportive). The relative change is obtained by comparing with the means shown in Appendix A, Tale A.5.

For the specific government responses, the poorest two quintiles are less supportive than the richest quintile in shutting public transportation, limiting mobility outside the home, and forbidding mass gatherings. The estimated coefficients are nearly always significant at the 5 percent level. Together, these results support our first hypothesis that poorer population groups were more likely to resist strict policies, voicing their disapproval, and shirking responsibilities under government orders.

Further unpacking the estimation results for each country, Table A.6 in Appendix A of S1 File shows that the countries, where individuals in poorer quintiles provided the least support for government responses include China, Italy, South Korea, and the U.S. However, while the estimation results for the poorer quintiles for Japan and the U.K. are still negative, they lose statistical significance. This is possibly due to the fact that these two countries have lower economic inequality than the others and Japan experienced a low COVID-19 infection rate.

**Fig 3:**
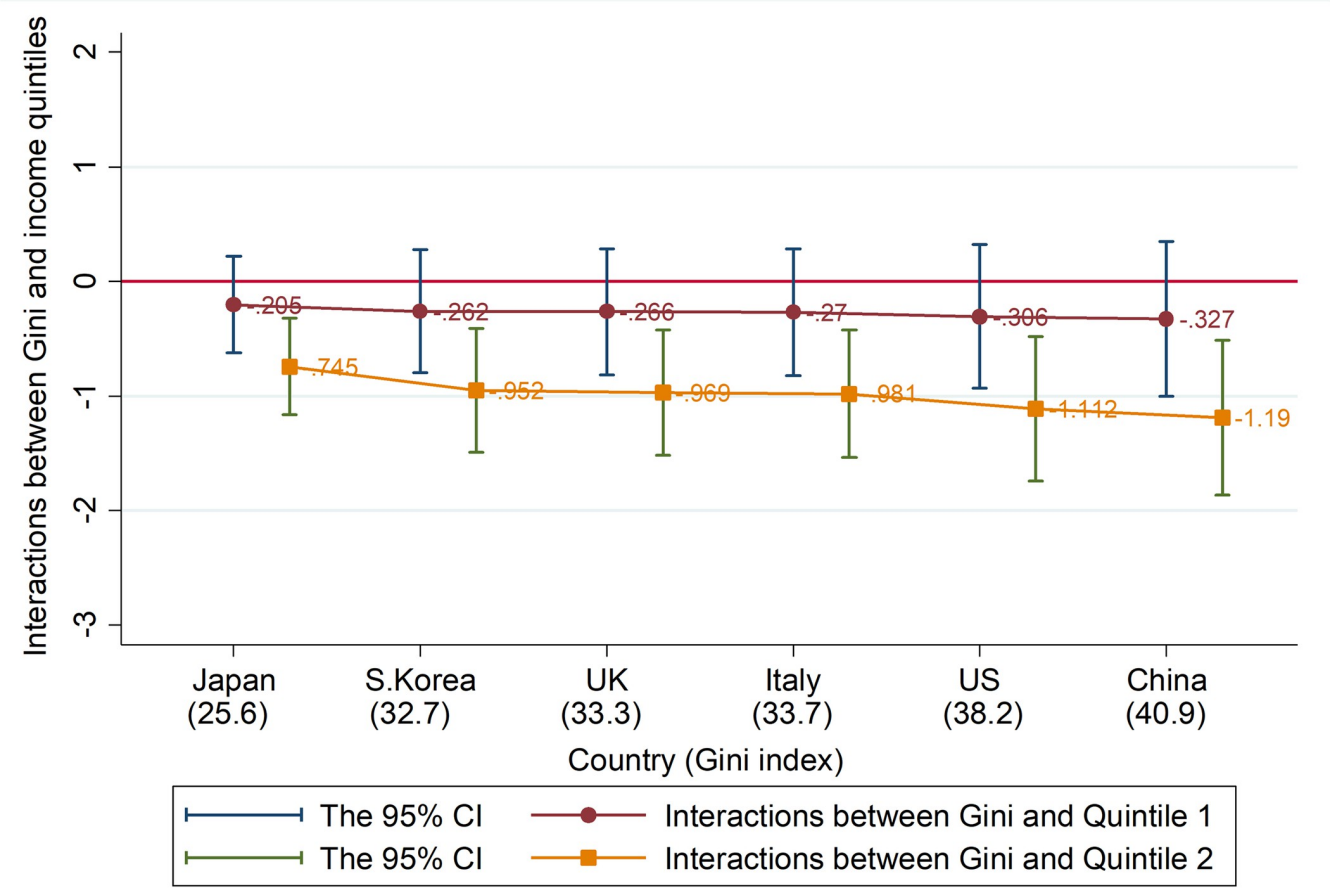
Estimated interactions between Gini index and income quintile. This figure presents the estimates of the interactions between Gini index and income quintiles in regressions of ‘Believe in the approach of the government in response to the pandemic’. The Gini index of each country is reported in parentheses. The full regression results using the pooled sample are reported in Table A.7 in Appendix A of S1 File.

To test the second hypothesis, we probe how inequality conditions the resistance of the poor to strict lockdown polices by interacting the income quintiles with a country’s inequality level. The estimation results, shown in Fig 3 (the full regressions reported in Table A.7 in the Appendix A of S1 File), suggest that in countries with high Gini indexes, the second poorest quintile is strongly less supportive of government responses. Yet, as discussed earlier in the Methods and Data section, the data do not allow clear-cut analysis for the two poorest quintiles in several countries, so this result may apply more generally for these two quintiles.

To focus our presentation, we only consider the overall assessment of government responses as our main dependent variable. But we also construct an aggregation index for the seven specific assessments using Principal Component Analysis (PCA) and alternative inequality measure from the World Development Indicator database as robustness checks. The results are qualitatively similar and somewhat statistically stronger (see Appendix A, Table A.7 in S1 File for full regression results).

While we acknowledge that more data points are needed to clearly prove the second hypothesis across different countries, the estimation results are supportive. We provide several additional robustness checks including potential multiple testing issues, alternative econometric models (including ordered logit models, different definitions of poorer income quintiles, and further controlling for country-level variables), and different measures of inequality and data source. These are discussed in more detail in Appendix B.

In Table A.8 in S1 File, we look at the heterogeneous association between income quintiles and assessment of the government responses across the employment sectors (including management and professionals, manufacture, tourism and accommodation, trade, and service). Overall, most interactions are not statistically significant, indicating the association between income quintiles and assessment of the government responses is quite similar across the employment sectors.

### Mediating role of economic and non-economic factors

To shed more light on the mechanisms leading to the effects, we test whether economic and non-economic factors mediate the relationship between income and support for government responses. We first regress individuals’ self-reported assessment of several economic and non-economic consequences on the income quintiles. Table A.9 in Appendix A of S1 File shows that poorer people report more adverse effects of the pandemic. We subsequently regress individuals’ overall assessment of the government response variable on their assessment of the economic and non-economic consequences. The results, offered in Table A.10 in Appendix A of S1 File, show that individuals are less likely to support government responses if they report more adverse effects of outcomes, such as (permanent or temporary) job losses, enjoying less free time, and feeling more bored.

Using the results from Tables A.9 and A.10 in S1 File, we employ a causal mediation approach to estimate the indirect relationship between the poorest income quintile and the overall assessment of government responses through several economic and non-economic outcomes that are caused by the pandemic. Recent evidence suggests that poorer quintiles are also found to experience reduced expected own-labor income, less savings, and are less likely to change their behaviors, both in terms of immediate prevention measures against COVID-19 and healthy activities [37, 38]. Put differently, the indirect impacts with these outcomes can help better explain the channels through which being in the poorest quintile leads to less support for government responses.

**Fig 4:**
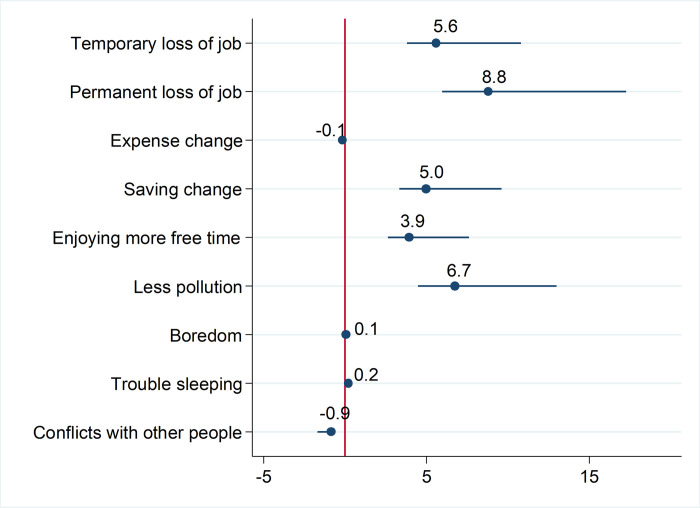
The proportion of the indirect impacts to the total impacts of being in the poorest quintile and overall assessment of government responses through several mediating variables (%) (point estimates and the 95% confidence interval). This figure presents the estimates of the indirect effects using “medeff” command in Stata [39].

Fig 4 shows the estimated shares (in percent) of the indirect impacts on the total impacts of being in the poorest quintile and overall assessment of government responses. The estimation results suggest that the potential for permanent job loss is the most important variable, accounting for 9 percent of the total effects. This is followed by the variables in the following order: less pollution (7 percent), temporary job loss (6 percent), less savings (5 percent), and enjoying more free time (4 percent). The remaining variables (i.e., expense change, boredom, trouble sleeping, and others) contribute very little to the total effects. These results provide supportive evidence for hypothesis 3 that while economic factors represent more burdens for poorer citizens, non-economic factors also play an important role.

## Discussion

We offer the first study that attempts to shed light on the complex relationship between inequality and support for government responses to the COVID-19 pandemic. Our findings using rich individual data from six countries (China, Italy, Japan, South Korea, the United Kingdom, and the United States) suggest that poorer individuals are likely less supportive of government responses, and poorest individuals are least supportive. Moreover, individuals in poorer quintiles residing in more economically unequal countries tend to offer even less government support.

Regarding the channels through which inequality can affect individuals’ government support, we find that economic and, to a lesser extent, non-economic factors play important roles. These include having greater job security, savings, free time, and better living environments (with less pollution).

We acknowledge that our study has several limitations. One limitation is that the results on the relationship between income quintiles and support for government responses should be interpreted as correlational rather than causal. Another challenge is a data constraint, where the Gini coefficient is at the country level and the individual income data are only available in income quintiles, so we can offer limited analysis. Nevertheless, our results concur with other studies in the U.S. showing that low-income households are less likely to work from home and to avoid large gatherings and public transportation or take health precautions [40, 41]. Furthermore, [42] even find a reversal in the ordering of social distancing by income where wealthy areas changed from most mobile before the pandemic to least mobile, but the poorest areas went from least mobile to most for multiple measures. Finally, since individuals are asked about the economic difficulties they experience before by their support for government policies (see the questionnaire sample in [33]), there can be some priming effects due to the order of questions (see, e.g., [43, 44]. In particular, the poorer individuals may be more likely to report that they do not support these policies. However, it remains unclear whether such priming effects exist in our context and if they do, the magnitudes of these effects are difficult to quantify. For example, while [45] find that overall satisfaction with public services is higher if questions about rating general satisfaction come first, [46] find the opposite result. There is clearly future scope for research on this topic.

Yet, despite these shortcomings, an optimistic interpretation of our results is that these factors are amenable to compensation policies. Governments can gather more support from the poorer population groups through social protection measures that better preserve employment, that offer more employee benefits, or that simply improve the living environment. For example, a recent review study suggests that negative economic shocks tend to increase support for redistribution and governmental cash transfers increase support for the incumbent, even if temporarily [47]. Offering more resources to the poor may also help reduce the potential deepening of social inequalities and reduced social trust caused by the pandemic.

## Supporting information

S1 File Online supplementary appendices A and B. (DOCX)

S1 Text (DOCX)
