# Supplementary material for: Inequality and support for government responses to COVID-19
Source: PLoS One. 2022 Sep 21;17(9):e0272972. doi: 10.1371/journal.pone.0272972 (PMC9491526; doi:10.1371/journal.pone.0272972)
Supplement: S1 File — (DOCX) [file pone.0272972.s001.docx]

# Appendix A: Additional Tables

## Table A.1. Comparison of gender and age of respondents of the survey and the national statistics

|  | China | | | Japan | | | South Korea | | | Italy | | | United Kingdom | | | United States | | |
| --- | --- | --- | --- | --- | --- | --- | --- | --- | --- | --- | --- | --- | --- | --- | --- | --- | --- | --- |
|  | Survey | Official | Diff. | Survey | Official | Diff. | Survey | Official | Diff. | Survey | Official | Diff. | Survey | Official | Diff. | Survey | Official | Diff. |
| **Gender** |  |  |  |  |  |  |  |  |  |  |  |  |  |  |  |  |  |  |
| % female respondents | 51.0 | 49.2 | 1.8 | 51.7 | 51.5 | 0.2 | 49.8 | 50.1 | -0.3 | 49.1 | 51.7 | -2.6 | 50.8 | 50.9 | -0.1 | 56.5 | 50.1 | -1.5*** |
|  |  |  | (1.6) |  |  | (1.6) |  |  | (1.6) |  |  | (1.5) |  |  | (1.6) |  |  | (1.5) |
| ***Distribution by age groups*** |  |  |  |  |  |  |  |  |  |  |  |  |  |  |  |  |  |  |
| % age between 18 and 25 | 15.1 | 13.3 | 1.8 | 10.5 | 10.6 | -0.1 | 13.4 | 12.7 | 0.7 | 11.4 | 11.1 | 0.3 | 10.7 | 13.9 | -3.2*** | 11.6 | 16.1 | 5.5*** |
|  |  |  | (1.1) |  |  | (1.0) |  |  | (1.1) |  |  | (1.0) |  |  | (1.0) |  |  | (1.0) |
| % age between 26 and 35 | 19.3 | 19.9 | -0.6 | 16.6 | 11.9 | 4.7*** | 17.7 | 15.1 | 2.6*** | 17.8 | 12.4 | 5.3*** | 17.4 | 16.4 | 1.0 | 17.4 | 17.4 | -1.2 |
|  |  |  | (1.3) |  |  | (1.2) |  |  | (1.2) |  |  | (1.2) |  |  | (1.2) |  |  | (1.2) |
| % age between 36 and 45 | 22.8 | 17.4 | 5.6*** | 16.0 | 15.0 | 1.0 | 19.6 | 17.2 | 2.4** | 20.0 | 14.9 | 5.1*** | 18.3 | 15.9 | 2.4** | 17.3 | 15.5 | 1.8 |
|  |  |  | (1.3) |  |  | (1.1) |  |  | (1.3) |  |  | (1.2) |  |  | (1.2) |  |  | (1.2) |
| % age between 46 and 55 | 18.4 | 20.9 | -2.5 | 20.5 | 16.6 | 3.9*** | 20.9 | 19.2 | 1.7 | 18.4 | 18.5 | -0.1 | 18.9 | 16.0 | 2.9** | 15.3 | 15.0 | 0.3 |
|  |  |  | (1.2) |  |  | (1.3) |  |  | (1.3) |  |  | (1.2) |  |  | (1.2) |  |  | (1.1) |
| % age between 56 and 65 | 12.8 | 14.5 | -1.7** | 16.7 | 13.9 | 2.8*** | 14.4 | 17.7 | -3.3*** | 15.1 | 16.4 | -1.3 | 18.8 | 15.1 | 3.7*** | 15.5 | 15.6 | -0.1 |
|  |  |  | (1.1) |  |  | (1.2) |  |  | (1.1) |  |  | (1.1) |  |  | (1.2) |  |  | (1.1) |
| % age between 66 and 75 | 7.7 | 9.4 | -1.7** | 13.3 | 16.0 | -2.7** | 11.9 | 10.4 | 1.5 | 15.0 | 13.2 | 1.8* | 13.0 | 12.1 | 0.9 | 14.2 | 11.9 | 2.3** |
|  |  |  | (0.8) |  |  | (1.1) |  |  | (1.0) |  |  | (1.1) |  |  | (1.1) |  |  | (1.1) |
| % age above 75 | 4.0 | 4.6 | -0.6 | 6.4 | 16.1 | -9.7*** | 2.1 | 7.6 | -5.5*** | 2.2 | 13.6 | -11.4*** | 2.9*** | 10.5 | -7.6*** | 8.7 | 8.5 | 0.2 |
|  |  |  | (0.6) |  |  | (0.8) |  |  | (0.5) |  |  | (0.5) |  |  | (0.5) |  |  | (0.9) |
| Total | 100 | 100 | 1.8 | 100 | 100 | 0.2 | 100 | 100 | -0.3 | 100 | 100 | -2.6 | 100 | 100 | -0.1 | 100 | 100 |  |
| Note: This table compares the proportion of respondents by gender and age of respondents, which are estimated by the COVID-19 survey, and the official number which are obtained from <https://www.populationpyramid.net/>.  The standard error of the differences is reported in parentheses. We assume that there are no standard errors associated with the official estimates.  *** p<0.01, ** p<0.05, * p<0.1: denote the significance level of the Z-test of equality of the proportion between the COVID-19 estimates and the official ones. | | | | | | | | | | | | | | | | | | |

## Table A.2. Income quintiles of respondents

| Income quintiles | China | Japan | South Korea | Italy | United Kingdom | United States | All the sample |
| --- | --- | --- | --- | --- | --- | --- | --- |
| First quintile | 20.2 | 21.1 | 21.5 | 16.7*** | 18.1* | 17.4** | 19.1* |
|  | (1.3) | (1.3) | (1.4) | (1.2) | (1.2) | (1.2) | (0.5) |
| Second quintile | 20.0 | 21.3 | 17.7* | 17.5** | 18.1* | 18.9 | 18.9** |
|  | (1.3) | (1.3) | (1.3) | (1.2) | (1.2) | (1.2) | (0.5) |
| Third quintile | 19.9 | 21.8 | 21.7 | 23.9** | 19.7 | 21.0 | 21.3** |
|  | (1.3) | (1.3) | (1.4) | (1.3) | (1.3) | (1.3) | (0.5) |
| Fourth quintile | 19.9 | 19.0 | 21.8 | 25.8*** | 22.3* | 23.6*** | 22.1** |
|  | (1.3) | (1.3) | (1.4) | (1.4) | (1.3) | (1.3) | (0.5) |
| Fifth quintile | 19.9 | 16.8* | 17.3** | 16.2*** | 21.8 | 19.2 | 18.5** |
|  | (1.3) | (1.2) | (1.2) | (1.2) | (1.3) | (1.2) | (0.5) |
| Total | 100 | 100 | 100 | 100 | 100 | 100 | 100 |
| Note: This tables reports the distribution of respondents by the income quintiles. Belot et al.’s (2020) COVID-19 survey only collects data on whether survey respondents fall into one of five income brackets, which are obtained by calculating quintiles of the gross household income distribution from the last available wave of nationally representative household surveys or census data. If the COVID-19 survey samples are representative for all the income quintiles, the proportion of respondents in each quintile should be 20%. The standard error of the estimates from the survey is reported in parentheses.  *** p<0.01, ** p<0.05, * p<0.1: denote the significance level of the Z-test of equality of the proportion between the COVID-19 estimates and 20%. | | | | | | | |

## Table A.3. Distribution of respondents by the level of belief in the responses to the COVID-19

|  | How effective do you believe each of these measures is in reducing the spread of the epidemic? | | | | | | | |
| --- | --- | --- | --- | --- | --- | --- | --- | --- |
|  | Shutting down schools | Shutting down public transport | Shutting down non-essential businesses | Limiting mobility outside home | Forbidding mass gatherings | Introducing fines for citizens that don't respect public safety measures | Requiring masks to be worn outside by everyone | Overall government response to the pandemic |
| Not effective at all | 4.5 | 5.7 | 4.9 | 4.4 | 3.7 | 6.6 | 7.1 | 6.1 |
| Slightly effective | 10.8 | 9.6 | 10.0 | 8.8 | 4.6 | 9.0 | 9.6 | 9.2 |
| Moderately effective | 18.4 | 21.3 | 22.4 | 20.1 | 11.8 | 20.6 | 18.6 | 20.5 |
| Very effective | 33.8 | 32.5 | 31.9 | 33.8 | 26.0 | 29.4 | 26.7 | 32.7 |
| Extremely effective | 32.6 | 31.0 | 30.8 | 33.0 | 53.9 | 34.5 | 38.0 | 31.6 |
| Total | 100.0 | 100.0 | 100.0 | 100.0 | 100.0 | 100.0 | 100.0 | 100.0 |

## Table A.4. Mean of outcomes and inequality variables

| Variables | China | Italy | Japan | South Korea | The UK | The US | All sample |
| --- | --- | --- | --- | --- | --- | --- | --- |
| Believe in the government in response to the pandemic | 4.52 | 3.73 | 2.90 | 3.68 | 3.89 | 3.76 | 3.74 |
| Believe in Shutting down schools | 4.00 | 4.11 | 3.42 | 3.68 | 3.51 | 4.01 | 3.79 |
| Believe in Shutting down public transport | 3.94 | 3.97 | 3.62 | 3.09 | 3.77 | 3.97 | 3.73 |
| Believe in Shutting down non-essential businesses | 4.10 | 3.97 | 3.59 | 3.08 | 3.78 | 3.86 | 3.74 |
| Believe in Limiting mobility outside home | 3.99 | 3.99 | 3.79 | 3.36 | 3.88 | 3.87 | 3.82 |
| Believe in Forbidding mass gatherings | 4.23 | 4.40 | 4.00 | 4.23 | 4.24 | 4.20 | 4.22 |
| Believe in Introducing fines for citizens that don't respect public safety measures | 3.97 | 4.00 | 3.44 | 3.90 | 3.67 | 3.59 | 3.76 |
| Believe in Requiring masks to be worn outside by everyone | 4.34 | 4.04 | 3.55 | 4.14 | 2.88 | 3.80 | 3.79 |
| Gini index (in percent) (Solt database) | 40.9 | 33.7 | 25.6 | 32.7 | 33.3 | 38.2 | 34.1 |
| Gini index (in percent) (World Development Indicators) | 38.5 | 35.9 | 32.9 | 31.6 | 34.8 | 41.4 | 35.9 |
| Ratio 90^th^/10^th^ | 10.85 | 14.05 | 9.10 | 9.15 | 9.57 | 17.94 | 11.86 |
| Ratio 80^th^/20^th^ | 6.97 | 7.02 | 5.34 | 5.34 | 5.93 | 9.18 | 6.66 |
| The rate of COVID-19 cases per 1000 people | 0.05 | 1.73 | 0.13 | 0.21 | 1.53 | 7.28 | 1.88 |

## Table A.5. OLS regressions of belief that government’s policy to the COVID-19 pandemic was effective

| Explanatory variables | (1) | (2) | (3) | (4) | (5) | (6) | (7) | (8) |
| --- | --- | --- | --- | --- | --- | --- | --- | --- |
|  | Overall government response to the pandemic | Shutting down schools | Shutting down public transport | Shutting down non-essential businesses | Limiting mobility outside home | Forbidding mass gatherings | Introducing fines for citizens that don't respect public safety measures | Requiring masks to be worn outside by everyone |
| Poorest quintile | -0.1783*** | 0.0091 | -0.1153** | -0.0402 | -0.1049** | -0.1927*** | -0.0768 | -0.0800 |
|  | (0.0450) | (0.0480) | (0.0481) | (0.0474) | (0.0473) | (0.0455) | (0.0518) | (0.0498) |
| Second poorest quintile | -0.1324*** | -0.0323 | -0.1464*** | -0.0639 | -0.0806* | -0.1583*** | -0.0063 | -0.0797 |
|  | (0.0450) | (0.0477) | (0.0485) | (0.0475) | (0.0468) | (0.0446) | (0.0503) | (0.0493) |
| Middle quintile | -0.1450*** | 0.0566 | -0.0367 | 0.0420 | 0.0335 | -0.0633 | 0.0407 | 0.0650 |
|  | (0.0447) | (0.0449) | (0.0452) | (0.0449) | (0.0443) | (0.0416) | (0.0477) | (0.0470) |
| Second richest quintile | -0.0895** | 0.0290 | 0.0099 | 0.0485 | 0.0224 | -0.0377 | 0.0353 | -0.0592 |
|  | (0.0438) | (0.0440) | (0.0435) | (0.0434) | (0.0439) | (0.0408) | (0.0466) | (0.0458) |
| Control variables | Yes | Yes | Yes | Yes | Yes | Yes | Yes | Yes |
| Constant | 3.7012*** | 3.8575*** | 3.9584*** | 3.7566*** | 3.7136*** | 4.1796*** | 3.4475*** | 3.8065*** |
|  | (0.0674) | (0.0694) | (0.0715) | (0.0703) | (0.0693) | (0.0682) | (0.0766) | (0.0723) |
| Observations | 5,950 | 5,950 | 5,950 | 5,950 | 5,950 | 5,950 | 5,950 | 5,950 |
| R-squared | 0.168 | 0.064 | 0.077 | 0.090 | 0.051 | 0.032 | 0.039 | 0.163 |
| Note: The control variables include age groups, gender, urban dummy, country dummies, and COVID-19 infection rates. The richest income quintile is the reference group. Robust standard errors in parentheses.  *** p<0.01, ** p<0.05, * p<0.1 | | | | | | | | |

## Table A.6. Regression of variable ‘believe in the government in response to the pandemic’ for different countries

| Explanatory variables | China | Italy | Japan | Korea | UK | US |
| --- | --- | --- | --- | --- | --- | --- |
| Poorest quintile | -0.1545* | -0.2531** | -0.1437 | -0.3542** | -0.0550 | -0.1976 |
|  | (0.0834) | (0.1103) | (0.1231) | (0.1427) | (0.1046) | (0.1224) |
| Second poorest quintile | -0.2744*** | -0.0862 | -0.0275 | 0.0039 | -0.0655 | -0.3441*** |
|  | (0.0772) | (0.1116) | (0.1195) | (0.1359) | (0.1034) | (0.1279) |
| Middle quintile | -0.3432*** | -0.1405 | 0.0041 | -0.0143 | -0.1717 | -0.2848** |
|  | (0.0882) | (0.1083) | (0.1183) | (0.1280) | (0.1095) | (0.1232) |
| Second richest quintile | -0.1053 | 0.0122 | -0.0906 | -0.0448 | -0.0885 | -0.1967* |
|  | (0.0661) | (0.1132) | (0.1201) | (0.1259) | (0.1014) | (0.1130) |
| Control variables | Yes | Yes | Yes | Yes | Yes | Yes |
| Constant | 4.7118*** | 3.1364*** | 3.5674*** | 4.1371*** | 3.7677*** | 3.4783*** |
|  | (0.0842) | (0.1452) | (0.1289) | (0.1696) | (0.1495) | (0.1568) |
| Observations | 994 | 982 | 919 | 1,021 | 995 | 1,039 |
| R-squared | 0.035 | 0.024 | 0.031 | 0.038 | 0.056 | 0.038 |
| Note: The control variables include age groups, gender, urban dummy, country dummies, and COVID-19 infection rates. The richest income quintile is the reference group. Robust standard errors in parentheses.  *** p<0.01, ** p<0.05, * p<0.1 | | | | | | |

## Table A.7: OLS regressions with interactions

| Explanatory variables | (1) | (2) | (3) | (4) |
| --- | --- | --- | --- | --- |
|  | Believe in the approach of the government in response to the pandemic | Index of the variables 'believe to different policies' | Believe in the approach of the government in response to the pandemic | Index of the variables 'believe to different policies' |
| Poorest quintile | 0.0967 | 0.3304 | 0.4094 | 0.1540 |
|  | (0.2942) | (0.3230) | (0.5058) | (0.4649) |
| Second poorest quintile | 0.8628*** | 0.3894 | 1.6277*** | 0.9897** |
|  | (0.2958) | (0.3160) | (0.5138) | (0.4613) |
| Middle quintile | 0.7084** | 0.9665*** | 1.3697*** | 1.0364** |
|  | (0.2990) | (0.3115) | (0.5123) | (0.4408) |
| Second richest quintile | 0.4210 | 0.0459 | 0.7137 | 0.1376 |
|  | (0.2945) | (0.3053) | (0.4964) | (0.4182) |
| Richest quintile | *Reference* |  |  |  |
| Poorest quintile * Gini index | -0.0080 | -0.0125 | -0.0163 | -0.0070 |
|  | (0.0084) | (0.0093) | (0.0140) | (0.0130) |
| Second poorest quintile * Gini index | -0.0291*** | -0.0141 | -0.0490*** | -0.0301** |
|  | (0.0084) | (0.0091) | (0.0142) | (0.0129) |
| Middle quintile* Gini index | -0.0250*** | -0.0278*** | -0.0422*** | -0.0283** |
|  | (0.0086) | (0.0090) | (0.0142) | (0.0123) |
| Second richest quintile * Gini index | -0.0149* | -0.0011 | -0.0223 | -0.0036 |
|  | (0.0083) | (0.0088) | (0.0137) | (0.0116) |
| Control variables | Yes | Yes | Yes | Yes |
| Constant | 3.7689*** | 0.0248 | 3.8581*** | 0.0644 |
|  | (0.0694) | (0.0665) | (0.0848) | (0.0788) |
| Observations | 5,950 | 5,950 | 5,950 | 5,950 |
| R-squared | 0.170 | 0.063 | 0.171 | 0.062 |
| Note: The control variables include age groups, gender, urban dummy, country dummies, and COVID-19 infection rates. The richest income quintile is the reference group. Columns (1) and (2) use the Gini index from the Solt database, and Columns (3) and (4) use the Gini index from the World Bank World Development Indicators database. Robust standard errors in parentheses  *** p<0.01, ** p<0.05, * p<0.1 | | | | |

## Table A.8. Regression of variable ‘believe in the government in response to the pandemic’ and Index of the variables 'believe to different policies' with interactions

| Explanatory variables | (1) | (2) | (3) | (4) | (5) | (6) | (7) | (8) | (9) | (10) |
| --- | --- | --- | --- | --- | --- | --- | --- | --- | --- | --- |
|  | Believe in approach  of the government in response to the pandemic | Index of the variables 'believe to different policies' | Believe in approach  of the government in response to the pandemic | Index of the variables 'believe to different policies' | Believe in approach  of the government in response to the pandemic | Index of the variables 'believe to different policies' | Believe in approach  of the government in response to the pandemic | Index of the variables 'believe to different policies' | Believe in approach  of the government in response to the pandemic | Index of the variables 'believe to different policies' |
| Poorest quintile * Management and professionals | 0.1386 | -0.1663 |  |  |  |  |  |  |  |  |
|  | (0.1947) | (0.1790) |  |  |  |  |  |  |  |  |
| Second poorest quintile * Management and professionals | 0.1262 | -0.3693** |  |  |  |  |  |  |  |  |
|  | (0.1615) | (0.1687) |  |  |  |  |  |  |  |  |
| Middle quintile * Management and professionals | 0.2793** | 0.0342 |  |  |  |  |  |  |  |  |
|  | (0.1308) | (0.1165) |  |  |  |  |  |  |  |  |
| Second Richest quintile * Management and professionals | -0.0143 | -0.1226 |  |  |  |  |  |  |  |  |
|  | (0.1269) | (0.1090) |  |  |  |  |  |  |  |  |
| Poorest quintile * Manufacture |  |  | -0.2239 | -0.4036*** |  |  |  |  |  |  |
|  |  |  | (0.1487) | (0.1485) |  |  |  |  |  |  |
| Second poorest quintile * Manufacture |  |  | -0.1251 | 0.0121 |  |  |  |  |  |  |
|  |  |  | (0.1249) | (0.1168) |  |  |  |  |  |  |
| Middle quintile * Manufacture |  |  | -0.1910* | -0.1716 |  |  |  |  |  |  |
|  |  |  | (0.1149) | (0.1116) |  |  |  |  |  |  |
| Second Richest quintile * Manufacture |  |  | -0.0444 | 0.0949 |  |  |  |  |  |  |
|  |  |  | (0.1070) | (0.0965) |  |  |  |  |  |  |
| Poorest quintile * Tourism & accommodation |  |  |  |  | 0.1420 | 0.2996 |  |  |  |  |
|  |  |  |  |  | (0.2119) | (0.2463) |  |  |  |  |
| Second poorest quintile * Tourism & accommodation |  |  |  |  | -0.0419 | 0.0865 |  |  |  |  |
|  |  |  |  |  | (0.2246) | (0.2501) |  |  |  |  |
| Middle quintile * Tourism & accommodation |  |  |  |  | 0.1704 | 0.2152 |  |  |  |  |
|  |  |  |  |  | (0.2183) | (0.2188) |  |  |  |  |
| Second Richest quintile * Tourism & accommodation |  |  |  |  | 0.0686 | 0.1341 |  |  |  |  |
|  |  |  |  |  | (0.2183) | (0.2222) |  |  |  |  |
| Poorest quintile * Trade |  |  |  |  |  |  | 0.1776 | 0.2545 |  |  |
|  |  |  |  |  |  |  | (0.1687) | (0.1769) |  |  |
| Second poorest quintile * Trade |  |  |  |  |  |  | 0.1637 | 0.2183 |  |  |
|  |  |  |  |  |  |  | (0.1682) | (0.1581) |  |  |
| Middle quintile * Trade |  |  |  |  |  |  | 0.1917 | 0.2400* |  |  |
|  |  |  |  |  |  |  | (0.1644) | (0.1431) |  |  |
| Second Richest quintile * Trade |  |  |  |  |  |  | -0.0052 | -0.1150 |  |  |
|  |  |  |  |  |  |  | (0.1763) | (0.1617) |  |  |
| Poorest quintile * Service |  |  |  |  |  |  |  |  | -0.2220 | -0.0256 |
|  |  |  |  |  |  |  |  |  | (0.1374) | (0.1244) |
| Second poorest quintile * Service |  |  |  |  |  |  |  |  | -0.0579 | -0.0104 |
|  |  |  |  |  |  |  |  |  | (0.1073) | (0.1024) |
| Middle quintile * Service |  |  |  |  |  |  |  |  | -0.1813* | -0.0646 |
|  |  |  |  |  |  |  |  |  | (0.1044) | (0.0964) |
| Second Richest quintile * Service |  |  |  |  |  |  |  |  | -0.1138 | -0.1295 |
|  |  |  |  |  |  |  |  |  | (0.1006) | (0.0906) |
| Control variables | Yes | Yes | Yes | Yes | Yes | Yes | Yes | Yes | Yes | Yes |
| Constant | 3.7435*** | 0.0125 | 3.7061*** | 0.0137 | 3.7302*** | 0.0353 | 3.7373*** | 0.0404 | 3.6957*** | 0.0132 |
|  | (0.0728) | (0.0672) | (0.0736) | (0.0679) | (0.0721) | (0.0663) | (0.0723) | (0.0665) | (0.0743) | (0.0679) |
| Observations | 5,950 | 5,950 | 5,950 | 5,950 | 5,950 | 5,950 | 5,950 | 5,950 | 5,950 | 5,950 |
| R-squared | 0.175 | 0.065 | 0.174 | 0.066 | 0.174 | 0.065 | 0.174 | 0.066 | 0.175 | 0.064 |
| Note: The control variables include income quintile, age groups, gender, urban dummy, country dummies, and COVID-19 infection rates. The richest income quintile is the reference group.  Robust standard errors in parentheses  *** p<0.01, ** p<0.05, * p<0.1 | | | | | | | | | | |

## Table A.9. OLS regression of mediating variables on income quintiles

| Explanatory variables | Dependent variables | | | | | | | | |
| --- | --- | --- | --- | --- | --- | --- | --- | --- | --- |
|  | Temporary loss of job | Permanent loss of job | Expense change | Saving change | Enjoying more free time | Less pollution | Boredom | Trouble sleeping | Increased conflicts with other people |
| The rate of COVID-19 | 0.0005 | 0.0027** | -0.0050 | -0.0035 | 0.0023 | -0.0041*** | 0.0027* | 0.0029** | -0.0001 |
|  | (0.0008) | (0.0013) | (0.0035) | (0.0033) | (0.0015) | (0.0014) | (0.0015) | (0.0014) | (0.0008) |
| Poorest quintile | 0.0441*** | 0.0866*** | 0.0205 | -0.1329*** | -0.0509** | -0.0842*** | -0.0212 | 0.0069 | -0.0057 |
|  | (0.0099) | (0.0178) | (0.0518) | (0.0453) | (0.0198) | (0.0202) | (0.0211) | (0.0181) | (0.0119) |
| Second poorest quintile | 0.0232** | 0.0981*** | -0.0313 | -0.1800*** | -0.0155 | -0.0299 | -0.0299 | 0.0009 | 0.0210* |
|  | (0.0093) | (0.0180) | (0.0514) | (0.0449) | (0.0201) | (0.0204) | (0.0209) | (0.0179) | (0.0126) |
| Middle quintile | 0.0062 | 0.0805*** | -0.0475 | -0.1481*** | -0.0126 | -0.0120 | 0.0174 | -0.0029 | -0.0083 |
|  | (0.0083) | (0.0173) | (0.0501) | (0.0441) | (0.0195) | (0.0198) | (0.0203) | (0.0175) | (0.0115) |
| Second highest quintile | -0.0032 | 0.0445*** | -0.0696 | -0.0340 | -0.0148 | -0.0170 | -0.0125 | -0.0096 | 0.0074 |
|  | (0.0081) | (0.0169) | (0.0492) | (0.0435) | (0.0191) | (0.0195) | (0.0200) | (0.0172) | (0.0116) |
| Richest quintile | *Reference* |  |  |  |  |  |  |  |  |
| Control variables | Yes | Yes | Yes | Yes | Yes | Yes | Yes | Yes | Yes |
| Constant | 0.0736*** | 0.2048*** | 2.6319*** | 2.7517*** | 0.5891*** | 0.4811*** | 0.6891*** | 0.4083*** | 0.1623*** |
|  | (0.0154) | (0.0269) | (0.0756) | (0.0695) | (0.0297) | (0.0302) | (0.0299) | (0.0279) | (0.0196) |
| Observations | 5,950 | 5,950 | 5,950 | 5,950 | 5,950 | 5,950 | 5,950 | 5,950 | 5,950 |
| R-squared | 0.029 | 0.066 | 0.023 | 0.031 | 0.109 | 0.108 | 0.050 | 0.074 | 0.023 |
|  |  |  |  |  |  |  |  |  |  |
| Note: The control variables include age groups, gender, urban dummy, country dummies. Robust standard errors in parentheses  *** p<0.01, ** p<0.05, * p<0.1 | | | | | | | | | |

## Table A.10. OLS regression of 'Believe in the approach of the government in response to the pandemic' on mediating variables

| Explanatory variables | Dependent variable is 'Believe in the approach of the government in response to the pandemic' | | | | | | | | |
| --- | --- | --- | --- | --- | --- | --- | --- | --- | --- |
|  | Model 1 | Model 2 | Model 3 | Model 4 | Model 5 | Model 6 | Model 7 | Model 8 | Model 9 |
| The rate of COVID-19 | 0.0046 | 0.0050 | 0.0046 | 0.0047 | 0.0042 | 0.0051 | 0.0045 | 0.0047 | 0.0044 |
|  | (0.0035) | (0.0035) | (0.0035) | (0.0035) | (0.0035) | (0.0035) | (0.0035) | (0.0035) | (0.0035) |
| Poorest quintile | -0.1683*** | -0.1625*** | -0.1786*** | -0.1695*** | -0.1713*** | -0.1663*** | -0.1782*** | -0.1779*** | -0.1796*** |
|  | (0.0450) | (0.0452) | (0.0450) | (0.0451) | (0.0450) | (0.0449) | (0.0450) | (0.0450) | (0.0450) |
| Second poorest quintile | -0.1272*** | -0.1145** | -0.1319*** | -0.1205*** | -0.1303*** | -0.1282*** | -0.1322*** | -0.1324*** | -0.1275*** |
|  | (0.0449) | (0.0450) | (0.0450) | (0.0449) | (0.0450) | (0.0449) | (0.0450) | (0.0449) | (0.0450) |
| Middle quintile | -0.1436*** | -0.1303*** | -0.1442*** | -0.1351*** | -0.1432*** | -0.1433*** | -0.1451*** | -0.1451*** | -0.1469*** |
|  | (0.0447) | (0.0446) | (0.0447) | (0.0446) | (0.0446) | (0.0446) | (0.0447) | (0.0447) | (0.0447) |
| Second highest quintile | -0.0902** | -0.0814* | -0.0884** | -0.0872** | -0.0874** | -0.0871** | -0.0894** | -0.0901** | -0.0877** |
|  | (0.0437) | (0.0437) | (0.0438) | (0.0437) | (0.0437) | (0.0436) | (0.0438) | (0.0437) | (0.0437) |
| Richest quintile | Reference |  |  |  |  |  |  |  |  |
| Temporary loss of job | -0.2276*** |  |  |  |  |  |  |  |  |
|  | (0.0696) |  |  |  |  |  |  |  |  |
| Permanent loss of job |  | -0.1827*** |  |  |  |  |  |  |  |
|  |  | (0.0335) |  |  |  |  |  |  |  |
| Expense change |  |  | 0.0158 |  |  |  |  |  |  |
|  |  |  | (0.0124) |  |  |  |  |  |  |
| Saving change |  |  |  | 0.0664*** |  |  |  |  |  |
|  |  |  |  | (0.0138) |  |  |  |  |  |
| Enjoying more free time |  |  |  |  | 0.1367*** |  |  |  |  |
|  |  |  |  |  | (0.0297) |  |  |  |  |
| Less pollution |  |  |  |  |  | 0.1423*** |  |  |  |
|  |  |  |  |  |  | (0.0301) |  |  |  |
| Boredom |  |  |  |  |  |  | 0.0065 |  |  |
|  |  |  |  |  |  |  | (0.0283) |  |  |
| Trouble sleeping |  |  |  |  |  |  |  | -0.0615* |  |
|  |  |  |  |  |  |  |  | (0.0336) |  |
| Increased conflicts with other people |  |  |  |  |  |  |  |  | -0.2356*** |
|  |  |  |  |  |  |  |  |  | (0.0530) |
| Control variables | Yes | Yes | Yes | Yes | Yes | Yes | Yes | Yes | Yes |
| Constant | 3.7180*** | 3.7386*** | 3.6596*** | 3.5185*** | 3.6207*** | 3.6328*** | 3.6967*** | 3.7263*** | 3.7394*** |
|  | (0.0676) | (0.0679) | (0.0753) | (0.0766) | (0.0689) | (0.0685) | (0.0701) | (0.0690) | (0.0682) |
| Observations | 5,950 | 5,950 | 5,950 | 5,950 | 5,950 | 5,950 | 5,950 | 5,950 | 5,950 |
| R-squared | 0.170 | 0.173 | 0.169 | 0.172 | 0.171 | 0.172 | 0.168 | 0.169 | 0.172 |
| Note: The control variables include age groups, gender, urban dummy, country dummies. Robust standard errors in parentheses  *** p<0.01, ** p<0.05, * p<0.1 | | | | | | | | | |

# Appendix B: Robustness Checks

We briefly discuss next several additional robustness checks. First, there may be a multiple testing problem with the eight outcome variables. Thus, in addition to the p-value of the estimate, which is the false positive rate among all the results, as a robustness check, we compute the q-values which constitute the false positive rate among significant results (i.e., the false discovery rate). The p-values and q-values of the coefficients of the poorest and the second poorest quintiles for these outcomes, shown in Figure B.1 in Appendix B, suggests that the most significant p-values remain significant using the q-values.^[[1]](#footnote-1)^ Second, since the dependent variable is a categorical variable, we also estimate Equation (1) with the alternative ordered logit model as a robustness check. The estimation results, expressed in odd ratios in Table B.1, Appendix B, are qualitatively similar.^[[2]](#footnote-2)^

Third, we use an alternative definition of the poorer income quintiles, where we separately consider the poorest quintiles, the two poorest quintiles, the three poorest quintiles, the four poorer quintiles, and their interaction terms with the Gini index. Estimates, shown in Table B.2, Appendix B show that the interaction terms for the three poorest quintiles or the four poorer quintiles are strongly statistically significant for belief in the approach of the government in response to the pandemic (columns 3 and 4), and to a weaker extent for the PCA index variable (columns 7 and 8).^[[3]](#footnote-3)^

Fourth, instead of using the Gini index to measure inequality, we use two ratios of the 90^th^/10^th^ and 80^th^/20^th^ income percentiles as robustness checks (since the Solt database does not provide these indicators, we use data from the World Bank World Development Indicators). These ratios focus on the differences between certain income percentiles, so are less general than the Gini index that focuses on the whole income distribution. The results are statistically weaker for the regressions with inequality. Nevertheless, they generally indicate that individuals that support government responses less include those in the second poorest quintile who live in countries with more inequality (Table B.3, Appendix B).

Fifthly, we include additional individual-level control variables in regressions. We include a variable indicating race of individual (black or white). A problem with this variable is that information on this variable is available in only the sample of US. For other countries, information on this variable is not available. Thus we include the interaction between the black and white variables with the dummy variable for the US in the pooled sample of the six countries. The interpretation of this variable is only available for the US. We include housing dummies of respondents and industry of the main employment (classified into 20 industries based on the questionnaires). The regression results, which are reported in Table B.4 in the Appendix, are similar to the main results reported in Table A.1. It should be noted that we do not use the results from regressions with controlling for housing and employment for interpretation, since housing and employment can be affected by the pandemic, and the results can be biased.

Finally, a limitation of the country fixed-effects model (equation (1)) is that it cannot include country time-invariant characteristics such as GDP per capita, unemployment, and education levels. We estimate a pooled OLS model where we control for log of GDP per capita, unemployment rate, and the proportion of people aged 25 and older with secondary education achievement. These variables are for 2019 and are taken from the World Bank’s World Development Indicators database. The estimation results, shown in Table B.4, Appendix B, are qualitatively similar.

**Additional References**

1. Simes, R. J. 1986. An improved Bonferroni procedure for multiple tests of significance. *Biometrika* 73(3), 751–754.
2. Newson, R. B. (2011). Frequentist Q-values for multiple-test procedures. *Stata Journal* 10(4), 568-584.

## Table B.1. Ordered Logit Regressions: Odds Ratio

|  | (1) | (2) | (3) | (4) | (5) | (6) | (7) | (8) |
| --- | --- | --- | --- | --- | --- | --- | --- | --- |
| Explanatory variables | Believe in the government in response to the pandemic | Believe in shutting down schools | Believe in shutting down public transport | Believe in shutting down non-essential businesses | Believe in limiting mobility outside home | Believe in forbidding mass gatherings | Believe in introducing fines for citizens that don't respect public safety measures | Believe in requiring masks to be worn outside by everyone |
| Poorest quintile | 0.7177*** | 1.0818 | 0.8743* | 0.9602 | 0.8752* | 0.7377*** | 0.9622 | 0.8942 |
|  | (0.0564) | (0.0844) | (0.0684) | (0.0760) | (0.0687) | (0.0616) | (0.0767) | (0.0721) |
| Second poorest quintile | 0.7602*** | 0.9720 | 0.8333** | 0.9053 | 0.8877 | 0.7407*** | 1.0318 | 0.8647* |
|  | (0.0592) | (0.0748) | (0.0649) | (0.0713) | (0.0683) | (0.0611) | (0.0793) | (0.0685) |
| Middle quintile | 0.7654*** | 1.0972 | 0.9631 | 1.0574 | 1.0653 | 0.8732* | 1.0776 | 1.0792 |
|  | (0.0594) | (0.0802) | (0.0700) | (0.0782) | (0.0786) | (0.0697) | (0.0780) | (0.0827) |
| Second Richest quintile | 0.8356** | 1.0452 | 1.0260 | 1.0594 | 1.0527 | 0.9218 | 1.0580 | 0.8825* |
|  | (0.0632) | (0.0737) | (0.0723) | (0.0757) | (0.0770) | (0.0722) | (0.0745) | (0.0650) |
| Richest quintile | Reference |  |  |  |  |  |  |  |
| Constant cut1 | 0.0527*** | 0.0330*** | 0.0331*** | 0.0379*** | 0.0487*** | 0.0332*** | 0.1037*** | 0.0548*** |
|  | (0.0067) | (0.0044) | (0.0044) | (0.0051) | (0.0063) | (0.0047) | (0.0130) | (0.0069) |
| Constant cut2 | 0.1541*** | 0.1353*** | 0.1033*** | 0.1352*** | 0.1655*** | 0.0804*** | 0.2738*** | 0.1575*** |
|  | (0.0184) | (0.0165) | (0.0129) | (0.0167) | (0.0195) | (0.0106) | (0.0327) | (0.0189) |
| Constant cut3 | 0.5483*** | 0.3991*** | 0.3543*** | 0.5038*** | 0.5664*** | 0.2277*** | 0.8781 | 0.4955*** |
|  | (0.0627) | (0.0472) | (0.0427) | (0.0597) | (0.0647) | (0.0287) | (0.1028) | (0.0577) |
| Constant cut4 | 2.7862*** | 1.7983*** | 1.5146*** | 2.1510*** | 2.4672*** | 0.8030* | 3.0735*** | 1.7194*** |
|  | (0.3195) | (0.2105) | (0.1805) | (0.2539) | (0.2822) | (0.0993) | (0.3630) | (0.1996) |
| Control variables | Yes | Yes | Yes | Yes | Yes | Yes | Yes | Yes |
| Observations | 5,950 | 5,950 | 5,950 | 5,950 | 5,950 | 5,950 | 5,950 | 5,950 |
| R-squared | 0.0721 | 0.0258 | 0.0291 | 0.0344 | 0.0201 | 0.0130 | 0.0129 | 0.0572 |
| Note: This table reports odds ratio of income quintiles in ordered logit regression of the responses to different government’s measures to COVID-19. The control variables include age groups, gender, urban dummy, country dummies, and the rate of COVID-19 cases. The richest income quintile is the reference group. Robust standard errors in parentheses. *** p<0.01, ** p<0.05, * p<0.1. | | | | | | | | |

## Table B2. OLS regressions with interactions

| Explanatory variables | (1) | (2) | (3) | (4) | (5) | (6) | (7) | (8) |
| --- | --- | --- | --- | --- | --- | --- | --- | --- |
|  | Believe in the approach of the government in response to the pandemic | | | | PCA Index of the variables 'believe in different policies' | | | |
|  | Model 1 | Model 2 | Model 3 | Model 4 | Model 1 | Model 2 | Model 3 | Model 4 |
| Poorest quintile | -0.4164* |  |  |  | -0.0414 |  |  |  |
|  | (0.2192) |  |  |  | (0.2461) |  |  |  |
| Poorest quintile * Gini index | 0.0098 |  |  |  | -0.0012 |  |  |  |
|  | (0.0063) |  |  |  | (0.0071) |  |  |  |
| Two poorest quintiles |  | 0.1341 |  |  |  | -0.0187 |  |  |
|  |  | (0.1819) |  |  |  | (0.1963) |  |  |
| Two poorest quintiles * Gini index |  | -0.0058 |  |  |  | -0.0025 |  |  |
|  |  | (0.0052) |  |  |  | (0.0057) |  |  |
| Three poorest quintiles |  |  | 0.4000** |  |  |  | 0.5192*** |  |
|  |  |  | (0.1852) |  |  |  | (0.1959) |  |
| Three poorest quintiles * Gini index |  |  | -0.0144*** |  |  |  | -0.0169*** |  |
|  |  |  | (0.0053) |  |  |  | (0.0057) |  |
| Four poorer quintiles |  |  |  | 0.6253*** |  |  |  | 0.3678 |
|  |  |  |  | (0.2286) |  |  |  | (0.2475) |
| Four poorer quintiles * Gini index |  |  |  | -0.0214*** |  |  |  | -0.0118 |
|  |  |  |  | (0.0065) |  |  |  | (0.0072) |
| Control variables | Yes | Yes | Yes | Yes | Yes | Yes | Yes | Yes |
| Constant | 3.5876*** | 3.6062*** | 3.6655*** | 3.7296*** | -0.0420 | -0.0021 | 0.0227 | 0.0111 |
|  | (0.0604) | (0.0603) | (0.0622) | (0.0680) | (0.0560) | (0.0563) | (0.0582) | (0.0646) |
| Observations | 5,950 | 6,089 | 6,089 | 6,089 | 5,950 | 6,089 | 6,089 | 6,089 |
| R-squared | 0.167 | 0.167 | 0.169 | 0.169 | 0.059 | 0.061 | 0.061 | 0.059 |
| Note: This table reports OLS regression of the responses to government’s measures to COVID-19. The control variables include age groups, gender, urban dummy, country dummies, and COVID-19 infection rates. The richest income quintile is the reference group. Gini index is from the Solt database. Robust standard errors in parentheses.  *** p<0.01, ** p<0.05, * p<0.1 | | | | | | | | |

## Table B.3. OLS regressions with interactions between income quintiles and ratio of income percentiles

| Explanatory variables | (1) | (2) | (3) | (4) |
| --- | --- | --- | --- | --- |
|  | Believe in the approach of the government in response to the pandemic | Index of the variables 'believe to different policies' | Believe in the approach of the government in response to the pandemic | Index of the variables 'believe to different policies' |
| Poorest quintile | 0.0265 | 0.0875 | 0.1143 | 0.1400 |
|  | (0.1736) | (0.1623) | (0.2387) | (0.2214) |
| Second poorest quintile | 0.2547 | 0.1612 | 0.5956** | 0.3652* |
|  | (0.1764) | (0.1619) | (0.2439) | (0.2215) |
| Middle quintile | 0.1072 | 0.2485 | 0.4152* | 0.4572** |
|  | (0.1748) | (0.1526) | (0.2414) | (0.2103) |
| Second Richest quintile | 0.1533 | 0.0968 | 0.2934 | 0.1183 |
|  | (0.1686) | (0.1462) | (0.2340) | (0.2002) |
| Richest quintile | *Reference* |  |  |  |
| Poorest quintile * Ratio 90^th^/10^th^ | -0.0175 | -0.0158 |  |  |
|  | (0.0147) | (0.0136) |  |  |
| Second poorest quintile * Ratio 90^th^/10^th^ | -0.0329** | -0.0216 |  |  |
|  | (0.0151) | (0.0137) |  |  |
| Middle quintile* Ratio 90^th^/10^th^ | -0.0214 | -0.0194 |  |  |
|  | (0.0148) | (0.0127) |  |  |
| Second Richest quintile * Ratio 90^th^/10^th^ | -0.0205 | -0.0075 |  |  |
|  | (0.0141) | (0.0121) |  |  |
| Poorest quintile * Ratio 80^th^/20^th^ |  |  | -0.0440 | -0.0358 |
|  |  |  | (0.0356) | (0.0331) |
| Second poorest quintile * Ratio 80^th^/20^th^ |  |  | -0.1096*** | -0.0689** |
|  |  |  | (0.0366) | (0.0334) |
| Middle quintile * Ratio 80^th^/20^th^ |  |  | -0.0843** | -0.0659** |
|  |  |  | (0.0362) | (0.0315) |
| Second Richest quintile * Ratio 80^th^/20^th^ |  |  | -0.0575* | -0.0166 |
|  |  |  | (0.0346) | (0.0297) |
| Control variables | Yes | Yes | Yes | Yes |
| Constant | 3.8278*** | 0.0689 | 3.8675*** | 0.0855 |
|  | (0.0959) | (0.0852) | (0.0937) | (0.0849) |
| Observations | 5,950 | 5,950 | 5,950 | 5,950 |
| R-squared | 0.169 | 0.061 | 0.170 | 0.062 |
| Note: This table reports OLS regression of the responses to government’s measures to COVID-19. The control variables include age groups, gender, urban dummy, country dummies, and COVID-19 infection rates. The richest income quintile is the reference group. The income ratio is from the World Bank’s World Development Indicators. Robust standard errors in parentheses.  *** p<0.01, ** p<0.05, * p<0.1 | | | | |

## Table B4. OLS regressions of agreement with government’s responses to the COVID-19 pandemic with controlling for additional individual-level observable variables

| Explanatory variables | (1) | (2) | (3) | (4) | (5) | (6) | (7) | (8) |
| --- | --- | --- | --- | --- | --- | --- | --- | --- |
|  | Overall government response to the pandemic | Shutting down schools | Shutting down public transport | Shutting down non-essential businesses | Limiting mobility outside home | Forbidding mass gatherings | Introducing fines for citizens that don't respect public safety measures | Requiring masks to be worn outside by everyone |
| Poorest quintile (Q1) | -0.1597*** | -0.0273 | -0.1100** | -0.0433 | -0.0951* | -0.2268*** | -0.0953* | -0.0762 |
|  | (0.0480) | (0.0511) | (0.0514) | (0.0506) | (0.0504) | (0.0488) | (0.0554) | (0.0535) |
| Second poorest quintile (Q2) | -0.1180*** | -0.0468 | -0.1296*** | -0.0587 | -0.0680 | -0.1724*** | -0.0148 | -0.0689 |
|  | (0.0456) | (0.0487) | (0.0497) | (0.0487) | (0.0481) | (0.0460) | (0.0518) | (0.0505) |
| Middle quintile (Q3) | -0.1301*** | 0.0478 | -0.0253 | 0.0462 | 0.0462 | -0.0667 | 0.0350 | 0.0788 |
|  | (0.0450) | (0.0459) | (0.0461) | (0.0459) | (0.0453) | (0.0427) | (0.0490) | (0.0479) |
| Second richest quintile (Q4) | -0.0757* | 0.0319 | 0.0227 | 0.0542 | 0.0325 | -0.0363 | 0.0343 | -0.0448 |
|  | (0.0439) | (0.0443) | (0.0441) | (0.0439) | (0.0443) | (0.0412) | (0.0470) | (0.0460) |
| Richest quintile (Q5) | Reference |  |  |  |  |  |  |  |
| The rate of COVID-19 | 0.0042 | 0.0046 | 0.0049 | 0.0063* | 0.0068** | 0.0053* | 0.0108*** | 0.0049 |
|  | (0.0035) | (0.0034) | (0.0033) | (0.0033) | (0.0034) | (0.0031) | (0.0040) | (0.0036) |
| Female respondents (female=1) | -0.0405 | -0.0990*** | -0.1393*** | -0.1552*** | -0.1552*** | -0.1365*** | -0.1281*** | -0.1622*** |
|  | (0.0289) | (0.0300) | (0.0301) | (0.0296) | (0.0294) | (0.0283) | (0.0320) | (0.0311) |
| Black (yes=1) | 0.2031 | -0.0438 | 0.2460* | 0.1065 | 0.1502 | 0.0364 | -0.1495 | 0.0019 |
|  | (0.1378) | (0.1277) | (0.1402) | (0.1413) | (0.1389) | (0.1345) | (0.1507) | (0.1432) |
| White (yes=1) | 0.1040 | -0.2118 | 0.0663 | 0.0844 | 0.1078 | -0.1223 | -0.2154 | -0.1231 |
|  | (0.1711) | (0.1673) | (0.1738) | (0.1721) | (0.1648) | (0.1687) | (0.1833) | (0.1758) |
| Age group (18 to 25) | Reference |  |  |  |  |  |  |  |
| Age group (26 to 35) | 0.1278** | 0.1716*** | 0.1187** | 0.1988*** | 0.1963*** | 0.1771*** | 0.1717*** | 0.0873 |
|  | (0.0498) | (0.0572) | (0.0579) | (0.0561) | (0.0566) | (0.0564) | (0.0599) | (0.0585) |
| Age group (36 to 45) | 0.1087** | 0.1722*** | 0.1355** | 0.1140** | 0.1241** | 0.1057* | 0.0446 | 0.0281 |
|  | (0.0499) | (0.0562) | (0.0565) | (0.0557) | (0.0556) | (0.0565) | (0.0591) | (0.0575) |
| Age group (46 to 55) | 0.1424*** | 0.2271*** | 0.1270** | 0.1452** | 0.2036*** | 0.1473*** | 0.0447 | -0.0823 |
|  | (0.0509) | (0.0562) | (0.0575) | (0.0564) | (0.0561) | (0.0561) | (0.0600) | (0.0576) |
| Age group (56 to 65) | 0.2094*** | 0.2290*** | 0.1253** | 0.1613*** | 0.3015*** | 0.2546*** | 0.1561*** | -0.0066 |
|  | (0.0530) | (0.0573) | (0.0587) | (0.0573) | (0.0570) | (0.0561) | (0.0604) | (0.0592) |
| Age group (66 to 75) | 0.2924*** | 0.2129*** | 0.1599*** | 0.1646*** | 0.2978*** | 0.2918*** | 0.1679** | 0.1481** |
|  | (0.0579) | (0.0605) | (0.0607) | (0.0600) | (0.0608) | (0.0575) | (0.0653) | (0.0619) |
| Age group (Above 75) | 0.2695*** | 0.1246 | 0.0824 | 0.1291 | 0.2199*** | 0.1763** | 0.1436 | -0.0506 |
|  | (0.0789) | (0.0822) | (0.0822) | (0.0825) | (0.0812) | (0.0771) | (0.0905) | (0.0864) |
| House | 0.0206 | 0.0145 | -0.0318 | -0.0082 | -0.0177 | 0.0227 | 0.0195 | -0.0082 |
|  | (0.0364) | (0.0381) | (0.0383) | (0.0371) | (0.0373) | (0.0363) | (0.0404) | (0.0391) |
| Apartment | 0.0234 | 0.0206 | 0.0329 | 0.0638 | 0.0094 | 0.0629 | 0.0437 | 0.0725* |
|  | (0.0408) | (0.0430) | (0.0429) | (0.0430) | (0.0426) | (0.0417) | (0.0460) | (0.0438) |
| Condominium | 0.0590 | -0.1062 | -0.2724 | -0.1480 | -0.2052 | -0.2508* | -0.0887 | -0.2123 |
|  | (0.1221) | (0.1644) | (0.1721) | (0.1498) | (0.1456) | (0.1508) | (0.1729) | (0.1684) |
| Trailer | -0.2138 | -0.3179 | -0.4243 | -0.3805 | -0.7423*** | -0.7691*** | -0.4763 | -0.3982 |
|  | (0.2758) | (0.2576) | (0.2662) | (0.2779) | (0.2646) | (0.2981) | (0.3494) | (0.2567) |
| Shelter | -0.1234 | 0.1051 | -0.2597* | -0.1011 | -0.0305 | 0.0472 | 0.1230 | -0.2321* |
|  | (0.1730) | (0.1298) | (0.1521) | (0.1471) | (0.1477) | (0.1377) | (0.1384) | (0.1330) |
| Office | -0.0383 | -0.0621 | -0.2172** | -0.1621 | -0.1662* | 0.0584 | -0.0043 | -0.1492 |
|  | (0.0945) | (0.1004) | (0.1092) | (0.0995) | (0.0976) | (0.0925) | (0.1037) | (0.1012) |
| Other housing types | Reference |  |  |  |  |  |  |  |
| Urban people (yes=1) | -0.0250 | -0.0070 | -0.0585* | 0.0021 | 0.0126 | -0.0528* | 0.0463 | 0.0927*** |
|  | (0.0312) | (0.0318) | (0.0322) | (0.0312) | (0.0314) | (0.0301) | (0.0340) | (0.0328) |
| Employment industry fixed-effects | Yes | Yes | Yes | Yes | Yes | Yes | Yes | Yes |
| Country fixed-effects | Yes | Yes | Yes | Yes | Yes | Yes | Yes | Yes |
| Constant | 3.5497*** | 3.9901*** | 3.8118*** | 3.7007*** | 3.6130*** | 4.2629*** | 3.6240*** | 3.8551*** |
|  | (0.1451) | (0.1342) | (0.1490) | (0.1490) | (0.1457) | (0.1428) | (0.1566) | (0.1492) |
| Observations | 5,950 | 5,950 | 5,950 | 5,950 | 5,950 | 5,950 | 5,950 | 5,950 |
| R-squared | 0.175 | 0.070 | 0.084 | 0.094 | 0.056 | 0.043 | 0.042 | 0.169 |
| Note: The richest income quintile is the reference group. The employment industries include accommodation and food services, administrative and support services, agriculture, art and entertainment, construction, finance and insurance, government, healthcare, information, management of companies, manufacturing, mining and quarrying, other services, professional and technical, real estate, retail trade, transportation, utilities, and wholesale trade.  Heteroskedasticity-robust standard errors. Log of GDP per capita, unemployment rate, and the proportion of people aged 25 and older with secondary education achievement are for 2019 and are taken from the World Bank’s World Development Indicators database.  *** p<0.01, ** p<0.05, * p<0.1 | | | | | | | | |

## Table B5. OLS regressions of agreement with government’s responses to the COVID-19 pandemic with controlling for country-level observable variables

| Explanatory variables | (1) | (2) | (3) | (4) | (5) | (6) | (7) | (8) |
| --- | --- | --- | --- | --- | --- | --- | --- | --- |
|  | Overall government response to the pandemic | Shutting down schools | Shutting down public transport | Shutting down non-essential businesses | Limiting mobility outside home | Forbidding mass gatherings | Introducing fines for citizens that don't respect public safety measures | Requiring masks to be worn outside by everyone |
| Poorest quintile (Q1) | -0.1971*** | 0.0235 | -0.1203** | -0.0489 | -0.1124** | -0.1963*** | -0.0780 | -0.0285 |
|  | (0.0454) | (0.0482) | (0.0481) | (0.0475) | (0.0472) | (0.0455) | (0.0518) | (0.0520) |
| Second poorest quintile (Q2) | -0.1497*** | -0.0191 | -0.1510*** | -0.0719 | -0.0875* | -0.1615*** | -0.0075 | -0.0323 |
|  | (0.0453) | (0.0479) | (0.0485) | (0.0475) | (0.0468) | (0.0446) | (0.0503) | (0.0513) |
| Middle quintile (Q3) | -0.1646*** | 0.0716 | -0.0420 | 0.0328 | 0.0256 | -0.0670 | 0.0394 | 0.1189** |
|  | (0.0448) | (0.0452) | (0.0452) | (0.0448) | (0.0443) | (0.0415) | (0.0477) | (0.0488) |
| Second richest quintile (Q4) | -0.1014** | 0.0381 | 0.0067 | 0.0430 | 0.0176 | -0.0400 | 0.0345 | -0.0265 |
|  | (0.0440) | (0.0443) | (0.0435) | (0.0433) | (0.0439) | (0.0408) | (0.0465) | (0.0484) |
| Richest quintile (Q5) | Reference |  |  |  |  |  |  |  |
| The rate of COVID-19 | 0.0010 | 0.0072** | 0.0044 | 0.0049 | 0.0054 | 0.0039 | 0.0101*** | 0.0147*** |
|  | (0.0035) | (0.0033) | (0.0033) | (0.0033) | (0.0033) | (0.0031) | (0.0039) | (0.0036) |
| Female respondents (female=1) | -0.0446 | -0.1265*** | -0.1470*** | -0.1701*** | -0.1662*** | -0.1717*** | -0.1371*** | -0.1917*** |
|  | (0.0281) | (0.0290) | (0.0293) | (0.0287) | (0.0285) | (0.0274) | (0.0309) | (0.0311) |
| Age group (18 to 25) | Reference |  |  |  |  |  |  |  |
| Age group (26 to 35) | 0.1262** | 0.1465*** | 0.1079* | 0.1829*** | 0.1870*** | 0.1533*** | 0.1632*** | 0.0628 |
|  | (0.0493) | (0.0568) | (0.0571) | (0.0552) | (0.0558) | (0.0558) | (0.0589) | (0.0589) |
| Age group (36 to 45) | 0.1085** | 0.1500*** | 0.1399** | 0.1044* | 0.1161** | 0.0811 | 0.0359 | 0.0128 |
|  | (0.0495) | (0.0554) | (0.0556) | (0.0546) | (0.0547) | (0.0557) | (0.0582) | (0.0577) |
| Age group (46 to 55) | 0.1369*** | 0.2030*** | 0.1271** | 0.1329** | 0.1911*** | 0.1255** | 0.0306 | -0.0995* |
|  | (0.0502) | (0.0559) | (0.0570) | (0.0556) | (0.0553) | (0.0554) | (0.0592) | (0.0591) |
| Age group (56 to 65) | 0.2247*** | 0.2180*** | 0.1444** | 0.1622*** | 0.3045*** | 0.2603*** | 0.1539*** | -0.0311 |
|  | (0.0527) | (0.0568) | (0.0582) | (0.0564) | (0.0560) | (0.0555) | (0.0595) | (0.0608) |
| Age group (66 to 75) | 0.3192*** | 0.2647*** | 0.2122*** | 0.1969*** | 0.3231*** | 0.3613*** | 0.1869*** | 0.1905*** |
|  | (0.0560) | (0.0586) | (0.0593) | (0.0584) | (0.0583) | (0.0562) | (0.0628) | (0.0625) |
| Age group (Above 75) | 0.2758*** | 0.2164*** | 0.1315* | 0.1504* | 0.2328*** | 0.2642*** | 0.1654* | 0.0817 |
|  | (0.0773) | (0.0803) | (0.0799) | (0.0798) | (0.0781) | (0.0755) | (0.0867) | (0.0861) |
| Urban people (yes=1) | -0.0442 | 0.0049 | -0.0738** | -0.0046 | 0.0011 | -0.0585** | 0.0461 | 0.1584*** |
|  | (0.0304) | (0.0309) | (0.0312) | (0.0303) | (0.0305) | (0.0290) | (0.0330) | (0.0331) |
| Log of GDP per capita | -0.7253*** | 0.1969* | 1.9997*** | 1.9252*** | 1.3693*** | -0.3733*** | -0.9637*** | -1.2595*** |
|  | (0.1284) | (0.1167) | (0.1258) | (0.1194) | (0.1227) | (0.1123) | (0.1276) | (0.1146) |
| Gini index (in percent) | 0.0842*** | 0.0323*** | -0.0082* | -0.0099** | -0.0186*** | 0.0147*** | 0.0173*** | 0.0273*** |
|  | (0.0041) | (0.0044) | (0.0044) | (0.0045) | (0.0043) | (0.0043) | (0.0048) | (0.0046) |
| Unemployment rate (in percent) | 0.0348*** | 0.0492*** | -0.0775*** | -0.0816*** | -0.0578*** | 0.0594*** | 0.0943*** | 0.0923*** |
|  | (0.0094) | (0.0091) | (0.0094) | (0.0092) | (0.0093) | (0.0087) | (0.0098) | (0.0093) |
| Share of people aged 25+ with secondary education achievement | 0.0101*** | -0.0044* | -0.0441*** | -0.0454*** | -0.0326*** | 0.0077*** | 0.0165*** | 0.0187*** |
|  | (0.0027) | (0.0025) | (0.0027) | (0.0025) | (0.0026) | (0.0024) | (0.0027) | (0.0024) |
| Constant | 7.6328*** | 0.5391 | -13.6026*** | -12.7537*** | -7.5546*** | 6.8697*** | 11.6390*** | 14.3063*** |
|  | (1.1033) | (0.9986) | (1.0713) | (1.0196) | (1.0481) | (0.9561) | (1.1027) | (0.9867) |
| Observations | 5,950 | 5,950 | 5,950 | 5,950 | 5,950 | 5,950 | 5,950 | 5,950 |
| R-squared | 0.157 | 0.057 | 0.077 | 0.087 | 0.049 | 0.032 | 0.039 | 0.087 |
| Note: The control variables include age groups, gender, urban dummy, and COVID-19 infection rates. The richest income quintile is the reference group.  Heteroskedasticity-robust standard errors. Log of GDP per capita, unemployment rate, and the proportion of people aged 25 and older with secondary education achievement are for 2019 and are taken from the World Bank’s World Development Indicators database.  *** p<0.01, ** p<0.05, * p<0.1 | | | | | | | | |

## Figure B.1. P-value and Q-value of the effect of the poorest and second poorest income quintile on the agreement to the government’s response to the COVID-19 pandemic

|  |
| --- |
|  |
|  |
|  |

1. The p-value is adjusted so that the chance of finding a random significant effect is reasonably small. The q-values are computed by [1]’s methods, using the ‘qqvalue’ command in Stata [2]. [↑](#footnote-ref-1)
2. For example, the four poorer quintiles have lower odds of support for government responses than the richest quintiles, with their odds ranging from 0.28 to 0.23 lower for the three poorer quintiles, and 0.16 lower for the second richest quintile (column 1). [↑](#footnote-ref-2)
3. The interactions for the lowest income groups are statistically insignificant in Table B.2, which appears not to provide strong support for the second hypothesis. Yet, one possible reason is that since we have fewer observations for the interaction term—especially for the bottom two income quintiles—this may explain to some extent this loss of statistical significance. Indeed, Table B.2 shows that the interaction term is positive but statistically insignificant for the poorest quintile. While the interaction term is still statistically insignificant, it is negative for the two poorest quintiles. It becomes both negative and statistically significant for the poorest three quintiles and the four poorer quintiles. [↑](#footnote-ref-3)
