# Supplementary material for: Inequality and support for government responses to COVID-19
Source: PLoS One. 2022 Sep 21;17(9):e0272972. doi: 10.1371/journal.pone.0272972 (PMC9491526; doi:10.1371/journal.pone.0272972)
Supplement: S1 Text — (DOCX) [file pone.0272972.s002.docx]

All replication files (data and do files in Stata) are available from the Harvard Dataverse database (<https://doi.org/10.7910/DVN/RIKJAY>).
